# Supplementary material for: Patterns of Intron Gain and Loss in Fungi
Source: PLoS Biol. 2004 Nov 30;2(12):e422. doi: 10.1371/journal.pbio.0020422 (PMC532390; doi:10.1371/journal.pbio.0020422)
Supplement: Table S1 — Also available at http://genes.mit.edu/NielsenEtAl/. (4.3 MB ZIP). [file pbio.0020422.st001.zip › NielsenEtAl/html/957.html]

AN1065.1.NCU09203.1.MG09004.1.FG02797.1


```
 CLUSTAL W (1.82) Multiple Sequence Alignments - Introns Inserted


Sequence 1: NCU09203.1	430 aa
Sequence 2: FG02797.1	428 aa
Sequence 3: MG09004.1	422 aa
Sequence 4: AN1065.1	429 aa
Alignment Length: 464 aa
Number Identitical Residues: 106 aa
Alignment Score (without introns) 6490


MG09004.1 	MSDVES------ETAGSPRAPTK--DDDQIDDAGE-------------------DSDVLS
NCU09203.1	MSDAAS------PAGSPAAEPTEHRDEDQVNETHQDDGSNHGGNDEDNDGGNDKDSDVLS
FG02797.1 	MSERDS------PVGEHTKEPVNDGQEQEYEDAVAAADSDH-------------DSDALS
AN1065.1  	MSASPSRSPESKPAGNTPPIDVDVENEQNVDAEEAVQPPTPG------AGGDDADADDND
          	**   * :..:. ..  .   ..  :::: :       .  .       .... *:*  .

MG09004.1 	DIDE~GVFEEEYN--PDTARVE-ERDYEDIAKSLKASKRKRGEGEAPPKRKTQLKKRRTV
NCU09203.1	EIDE~NEFGD------DYGSRP-VDIDENVAMKLKA--RRKTTTETT--KKPKEGRRPKK
FG02797.1 	EVDE~DQFEDYDPETANIEDRP-VDIDEDVARTLKATKRKRTEGEAP--KKPREGRRDKK
AN1065.1  	AKPA0AQFEDFDPENVDIEDRPQLDIDEENLKLIGRHKRKRTEEEGTS-KRKKEGRRRKS
          	       * :   .. :     .    *:    :   .*::   * .. :: :  :* . 

MG09004.1 	EAAADDLGDAAGGSDDENGSRRQRKSRMAGDSRRAKAKEKSPEPEIDESTLTPEER~RKR
NCU09203.1	RSRGDDDVDAADD-DG---ERRPRKVRAEGE-RRARKEVEEQQAQQEE-NLTPDER~RRR
FG02797.1 	RRDRDEDVEMDDAEDGSKKSRRSRRVAVDGE-RRQKSKAATPEPENED-HLSPEER~RKR
AN1065.1  	RRYAEEEEGGSDREAG-------------GK----RRKSKKAELEEDEETLDPATR1RRR
          	.   ::     . . .             *.    : :    : : ::. * *  * *:*

MG09004.1 	ALLKAMDAAAKGRS-SRRRKKDEV0DLEEMADEEIAALKISMENACVADNKAREEGKPAI
NCU09203.1	ALERAIDAAVKNPT-KRRRKKDDI0DLEEETDEQIANLKVAMEKACVADNEAREQKQPAV
FG02797.1 	AIDRALDAAIKKPSGQKRKKKDEI0DLEAEIDDLLADLKVRMEGACQSDNQAREAGQPAL
AN1065.1  	ALDRAMDEALKKPTKRRARKQDGI0DLEQMADAEIEDMRKRMTHAAQMDAINRREGKPAM
          	*: :*:* * *  :  : :*:* : ***   *  :  ::  *  *.  *   *.  :**:

MG09004.1 	EKLKLLPKVVAMMNRNAIQHAILDPETNFLQSLRFFLEPLNDGSLPAYNIQREIFTALLR
NCU09203.1	HKLKLLPQVTAILNRTAIQDSVLDPEINFLQSVRYFLEPLNDGSLPAYNIQRAIMSALMK
FG02797.1 	HKLKLLPEVTAIMNRNNVQHEVLDPDTNFLQHVKFFLEPLNDGSLPAYNIQRDIFNALAK
AN1065.1  	HKLKMLPEVVSLLNRNQYVNSLVDPEINLLEAVKFFLEPLDDGSLPAYNIQRDLMTALGK
          	.***:**:*.:::**.   . ::**: *:*: :::*****:*********** ::.** :

MG09004.1 	LPVEKEALLSSGIGKVVLFYTKSKRPEPGVKRMAEKLIGEWSRPILKRTDDFRKRHIETR
NCU09203.1	LPINKDVLLSSGIGKVVVYYNKSKSPSADIKRDAERLLGEWSRLILKRTDDYKKRHIEMR
FG02797.1 	MNIEKEALLSSGIGKVVVFYTRSKKPEPSIKRIAQRLLGEWSRPILNRTDDYKKRQIETR
AN1065.1  	LPINKETLIASGIGKVVVFYTRSKRPEPGIKRMAERLLAEWTRPILQRSDDYSKRVYQEA
          	: ::*:.*::*******::*.:** *...:** *::*:.**:* **:*:**: **  :  

MG09004.1 	EFDYA2AAKQAQET~ASYGSSQAATLPSRPAGRSIFEEEIQKKLAPK--NPYRTAGTGLQ
NCU09203.1	EIDVG2AVKLGQRE~G---GSSQVTLTQRPAGKSRYEIERERALAPEVRNNNRARPVGLP
FG02797.1 	DYDYD2AAKMAQRQ~K---TNSQFSLSQRPAPNTK-EAERERLLAPS-RGNNSARMVNLP
AN1065.1  	EFDPT~YVLSPFLL2K-------ITSRVKSVQDSAAEARARELLPPR--LANRARAEISH
          	: *    .                :   :..  :  * . .. *.*       :      

MG09004.1 	KTYTVAPQSTYNAPGG-GDYRPMGSGGLDAFRRMTQ------KGKKK--
NCU09203.1	ASYTIAPKSTYIPGQAPTDHRPIGHSGHEAFRRMTQ------KGKGKR-
FG02797.1 	SSYTVAPQSTFMGSQG-SGHRPLGAGGMEAFRKMTQ------KSKKRAN
AN1065.1  	TSYTVVPKPTVVQESK--FARPLGASGEDRFRKMRARQIAATKGQGARR
          	 :**:.*:.*          **:* .* : **:*   . :::*.:
```
